# Out-of-pocket health expenditure and fairness in utilization of health care facilities in Cambodia in 2005 and 2010

**DOI:** 10.12688/f1000research.12801.1

**Published:** 2017-11-29

**Authors:** Koustuv Dalal, Olatunde Aremu, Gainel Ussatayeva, Animesh Biswas

**Affiliations:** 1 Centre for Injury Prevention and Safety Promotion (CIPSP), School of Health Sciences, Örebro University, Örebro, SE-701 82, Sweden; 2 School of Health Sciences, Birmingham City University, Birmingham, B15 3TN, UK; 3 Higher School of Public Health, Al-Farabi Kazakh National University, Almaty, Kazakhstan; 4 Centre for Injury Prevention and Research, Dhaka, Bangladesh

**Keywords:** Equity, Fairness, Out-of-pocket payment, Health Expenditure, Low-income countries, Cambodia

## Abstract

**Background:** Out-of-pocket (OOP) payments for health care are highly pervasive in several low-and-middle income countries. The Cambodian health system has envisaged massive repositioning of various health care financing to ensure equitable access to health care. This analysis examines catastrophic, economic, as well as fairness, impacts of OOP health care payments on households in Cambodia over time.

**Methods:** Data from two waves of a nationally representative household survey conducted in Cambodia (CDHS Surveys 2005 and 2010) were utilized. Healthcare utilizations based on economic status were compared during 2005 and 2010. Variables of interests were i) where care was sought and the instances of treatments, i.e. was treatment sought the first, second or third time; (ii) the mode of payment for treatment of the respondent or for any household member due to sickness or injury in the last 30 days prior to the survey period. Lorenz curves were applied to assess the degree of distribution of inequality in OOP expenditures between different income brackets.

**Results:** The findings revealed that there was inequality and unfairness in health care payments, and catastrophic spending is more common among the poor in Cambodia. The majority of people from poorer households experienced economic hardship and have taken to catastrophic health care spending through sales of personal possessions.

**Conclusion:** Based on the findings from this analysis, more attention is needed on effective financial protection for Cambodians to promote fairness. The government should increase spending on services being provided at public health care facilities to reduce ever increasing reliance on private sector providers. These approaches would go a long way to reduce the economic burden of care utilization among the poorest.

## Introduction

Globally, the main goal of a health care system is to have appropriate funding mechanisms for individuals to acquire care for preventive and curative health needs without deepening into poverty. There is a general consensus that ease of access to health care facilities has the potential to improve the health and wellbeing of individuals and increase the life expectancy of the entire population ^1, 2^. However, the pluralistic nature and differences in financing mechanisms of most health systems around the world means varied methods will be adopted by policy makers for health care provision. Revenue collection through general taxation and compulsory social health insurance, as well as individual private health insurance schemes, are among the most commonly used methods for funding health care provisions in high income nations ^3^. Sadly, in spite of the successes of these funding strategies in high income nations, inadequate planning and lack of political will has given rise to non-existence of universal financing mechanisms in low-and-middle-income countries (LMICs). In these countries, many individuals continue to rely on out-of-pocket (OOP) payments each time they access care, and as a result, are not shielded from economic hardship due to huge health care expenditures. Catastrophic health expenditure through OOP payments is a global phenomenon and can exist regardless of health care funding mechanisms. However, OOP payments constitute a significant component of health spending in most LMICs. According to a study investigating global estimates of catastrophic spending and OOP payments ^4^, approximately 150 million people suffer financial burden each year due to health care payments, and about 100 million are pushed into poverty because of OOP payments.

Paying for health care through OOP may prevent patients from seeking medical care when needed due to economic constraints. This is particularly common for people with lower incomes ^5^. Low income groups sometimes risk debt due to paying for health care ^6^. In Cambodia, as is the case with most countries in South East Asia ^6– 9^, excessive reliance on OOP payment for care is pervasive due to lack of universal health coverage ^10^. Cambodia is a country with a turbulent history, enduring genocide and societal collapse, but is now rapidly evolving and has managed to decrease poverty significantly ^11^. The repositioning of the Cambodian health system is aimed at preventing and controlling communicable and non-communicable diseases ^12, 13^. Although the health care system has also experienced progress, it still is in need of further development, as the majority of health expenditure comes from OOP payments and this usually benefits unregulated private health care ^14^. There was a health care system reform in 1996, and the National Charter on Health Financing was introduced allowing user fees in health care facilities ^15^. In conjunction with this charter on health financing, a health equity fund was introduced in 2009 to aid access to health care for the poor. However, the health equity fund has not been implemented on a national scale, but rather has been used in different ways in different districts ^16, 17^. Examining the impact of catastrophic health care spending on household impoverishment during the introduction of a new funding mechanism, such as health equity fund, over a period of time is of policy relevance. While several studies have been conducted to assess the impact of catastrophic expenditure on household impoverishment over a period of time in other countries in South East Asia ^6– 10^, no such study have been done in Cambodia.

The present study was conducted to bridge this research gap and examine, for the first time, trends in catastrophic OOP payment burden and equity in use of health care facilities among a nationally representative sample of Cambodian adults aged 15–49 across 611 communities. Specifically, this study aimed to: first, examine fairness in the distribution of cost burden among wealth strata and compare trends between 2005 and 2010; and second, characterise sources of OOP payment for treatment and compare the trends between 2005 and 2010. Understanding such comparisons would aid in further planning and future implementation of various health benefit packages aimed at alleviating high OOP payments for healthcare use in Cambodia.

## Methods

### Data sources

Secondary data analysis was conducted using publicly available individual and community level data from the Cambodian Demographic and Health Surveys (CDHS) of 2005 and 2010 ^18, 19^. These survey data were collected from nationally representative samples of households. Approval to use the data was granted by The DHS Program, Rockville, USA. DHS are a series of population-based surveys commonly conducted in most LMICs by in-country national agencies under the technical assistance of ICF Macro with financial support from USAID.

Briefly, the CHDS employs a two-stage sampling procedure, with the first stage involving the selection of primary sampling units (PSUs); these are probability proportional to size and represent the number of households within the PSU. The second stage uses a systematic technique to sample households from each of the selected PSUs units. The full details of the methods and procedure used in data collection in CDHS surveys is provided elsewhere ^19^. A total of 8578 weighted sample of adults from two surveys was distributed as follows, which were used for analysis: 2589 in 2005 survey; 5989 in 2010 survey

### Study variables

The outcome variables for this analysis were: (i) where care was sought and the instances of treatments, i.e. was treatment sought the first, second or third time; (ii) the mode of payment for treatment of the respondent or for any household member due to sickness or injury in the last 30 days prior to the survey period. First, to estimate the distribution of economic hardship among wealth groups, principal component analysis (PCA) was used ^20^. PCA was used to calculate an asset index for each household using the respondent response about possession of a set of household assets. Second, the resulting index based on economic hardship was then used to rank households into quintiles as poorest, poorer, middle, richer and richest ^18– 20^. Individuals who live below the poverty line are determined from their households in the poorest quintile ^18– 20^. The derived wealth quintiles was used as the only dependent variable in this analysis.

### Analysis

Descriptive statistics was adapted to characterise healthcare utilization by the respondents. Lorenz curves were applied to assess the degree of distribution of inequality in OOP expenditures. The Lorenz curve is a measure of the distribution of wealth within a population ^21– 23^. Briefly, the X axis on the curve denotes the percentile of the population distributed according to the characteristic under observation. The observed characteristic in this analysis is the economic status, as a proxy of income ^19, 20^. The ( *y*) ** value of the curve represents the exact proportion of the overall value of costs accrued to people that are no wealthier than a specified estimated percentile of the population. For better understanding, we have used three different costs. These are cost of the treatment, transport cost and total costs,

The Lorenz curve can be expressed mathematically as shown belows

$$L(y)=\frac{\int_{0}^{y}xdF(x)}{\mu }$$

Where *F* ( *y*) represents the cumulative distribution functions of ordered individuals and *μ* represents the average size. The Lorenz curve, in the simplest form, is usually interpreted as follows: if there is no difference among all individuals under observation, the Lorenz curve would be a straight diagonal line, called the line of equality. However, if there is an existence of inequality among the population, then the Lorenz curve would show a fall below the line of equality ^23^. All the analyses were conducted using STATA 13 software package.

## Results

A total of 5989 participants participated in the 2010 study. Overall, 92% sought health services for a first-time treatment in 2010 compared to 90% in 2005. For services sought for second treatments, there was a decrease from 29% in 2005 to 24% in 2010. Similar results were shown for third treatments; 40% in 2005 to 33% in 2010.

### Utilization of health care facilities based on economic status

Table 1 depicts the information of service utilization for the 1st, 2nd and 3 ^rd^ instances of treatments for illness or injury. In 2005, almost 83% of people belonging to the household 20% below the poverty line (i.e. poorest quintile) sought treatment at the 1 ^st^ instance compared with 95% of those from rich household. There is an increase in the proportion of people from the poorest strata seeking treatment at the 1 ^st^ instance in 2010 (i.e. from 83% to 90%), but the number of the richest seeking treatment at the 1 ^st^ instance between 2005 and 2010 remains the same. There is, however, a significant reduction in the percentage of both the poorest and the richest that sought treatment in the 2 ^nd^ and 3 ^rd^ instances between 2005 and 2010; for example, 28% of both the poorest and richest sought treatment at the 2 ^nd^ instance in 2005, by 2010, the percentage of poorest people seeking treatment for the 2 ^nd^ instance fell to 23%. The analysis also revealed a decline to 20% in 2010 for rich people seeking care. For the 3 ^rd^ instance and in 2005, more people, 45% from the poorest households used heath care more than their rich counterparts (35%). In 2010, whilst the percentage of rich people seeking care for the 3 ^rd^ instance remained unchanged from that of 2005 and much lowered compared to those from poorest households, there is a decline in the percentage of poorest people (34%) seeking care in 2010 compared with 2005. Almost 90% and 70% of people in both the 20% below (poorest quintile) and 20% above (poorer quintile) the poverty line have in 2005 and 2010, respectively, have used more health care at the 3 ^rd^ instance than their rich counterparts (33%) for both years.

**Table 1.** Comparison of health care utilization according to economic status, between 2005 & 2010.

	Service sought (First treatment)				Service sought (Second treatment)				Service sought (Third treatment)			
	2005		2010		2005		2010		2005		2010	
	N	n (%)	N	n (%)	N	n (%)	N	n (%)	N	n (%)	N	n (%)
Poorest	560	P=0.000 463 (83)	1291	P=0.000 1158 (90)	463	P=0.651 130 (28)	1158	P=0.002 265 (23)	130	P=0.358 58 (45)	265	P=0.547 89 (34)
Poorer	567	511 (90)	1226	1118 (91)	511	148 (29)	1118	292 (26)	148	65 (45)	292	101 (35)
Middle	552	495 (90)	1173	1086 (93)	495	157 (32)	1086	284 (26)	157	59 (38)	284	88 (31)
Richer	470	433 (92)	1220	1138 (93)	433	131 (30)	1138	256 (23)	131	48 (37)	255	74 (29)
Richest	440	419 (95)	1079	1024 (95)	419	116 (28)	1024	202 (20)	116	41 (35)	202	74 (35)
Total	2589	2321 (90)	5989	5524 (92)	2321	682 (29)	5524	1299 (24)	682	271 (40)	1298	423 (33)

There was an increase in services sought for 1st treatment among all groups, except for the richest group, who remained the same, between 2005 and 2010. For services sought for 2 ^nd^ treatment, there was a decrease in all groups. Regarding health care utilization for 3rd treatment, a similar decrease was observed; however this was not significant in 2005 or 2010.

### Utilization of public and private health care facilities

**Figure 1.**
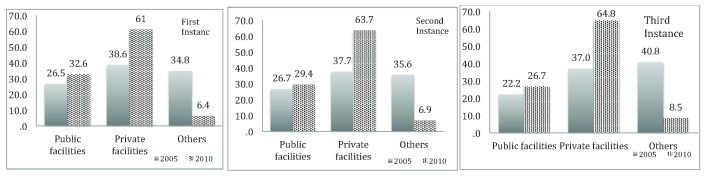
Utilization of public and private health care facilities in different instances.

Overall, there was an increase in utilization of both public and private health care facilities between 2005 and 2010, as can be seen in Figure 1. There was an increase in usage of private facilities as the preferred place for service utilization at 1st instance i.e. from 38.6% in 2005 to 61% in 2010. There was also an increase, albeit a small one, in use of public facilities, 26.5% in 2005 to 32.6% in 2010. For utilization of health care at the 2 ^nd^ instance, similar results can be found; increase in usage of private health care from 37.7% in 2005 to 63.7% in 2010 and increase in usage of public health care from 26.7% in 2005 to 29.4 in 2010. This is also the same for the 3 ^rd^ instance; an increase in utilization of private health care facilities from 37% in 2005 to 64.8% in 2010. There was been a decrease in usage of informal health care facilities (such as shop/market, magician, religious leader, all characterised as others), for all three different instances from 2005 to 2010. These results who that, generally, a higher percentage of Cambodians have a strong preference for private health care facilities.

### Households most likely to engage in catastrophic spending

**Figure 2.**
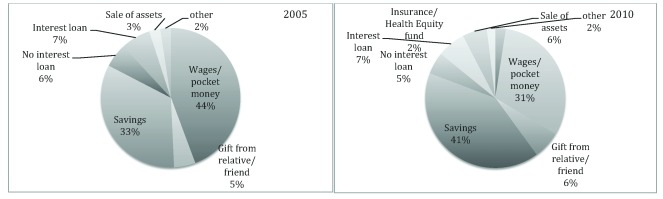
Mode of out-of-pocket payments for health care utilizations: comparison between 2005 and 2010.

***Mode of out-of-pocket payment.*** Figure 2 displays a breakdown of percentages of different modes of OOP. Briefly, the mode of OOP was divided into the following categories: wages/pocket money, savings, sale of assets, interest loan, non-interest loan, and other sources, e.g. such as help from an NGO. In 2005, 44% of all OOP payments came from wages/pocket money, followed by 33% from savings. In 2010, 31% of OOP payments were from wages/out of pocket money and 41% from savings. Payments from loans generally remained the same between 2005 and 2010, around 5-7%. Sale of assets as means to pay for OOP payments for health care increased from 3% in 2005 to 6% in 2010. Gifts from relatives and friends (5% in 2005 and 6% in 2010) were also OOP sources. The health equity fund introduced in 2009 seems to have effect on payments and accounted for 2% of the OOP payments in 2010.

**Figure 3.**
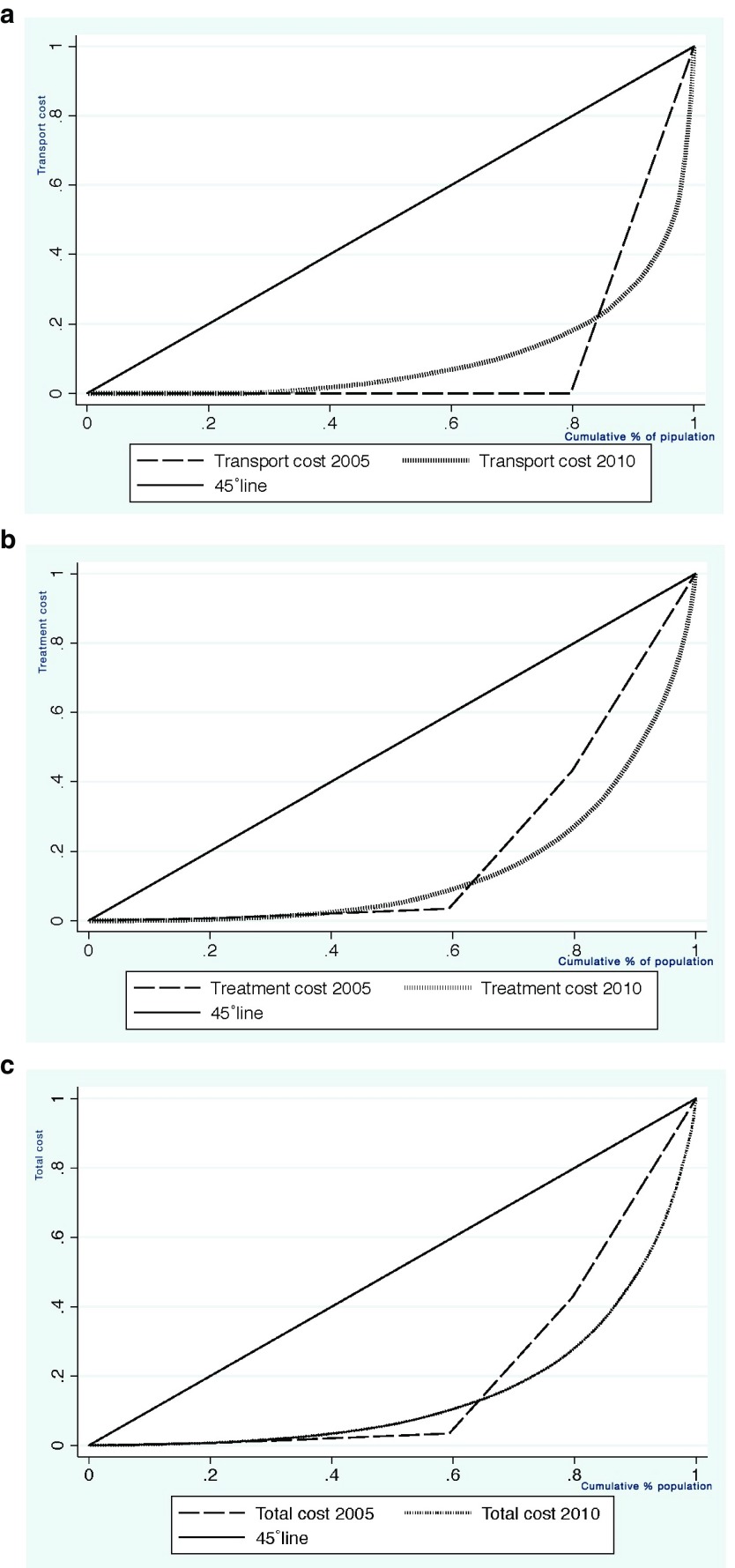
Lorenz curves for Transport cost ( **3a**), Treatment cost ( **3b**) and Total cost of treatment ( **3c**): comparison between 2005 and 2010.

***Transport cost, treatment cost and total cost of treatment.*** Degree of fairness in terms of spending on transport, treatment and total cost of treatment was examined using Lorenz curves ( Figures 3a–c). As depicted in Figure 3a, for all categories of treatment instances, the transportation cost is the most equal across nearly all the economic strata, with individuals in the poorest quintiles of the economic strata being more able to cope with spending. Compared to 2005, in 2010 individuals in the 3 ^rd^, 4 ^th^ and 5 ^th^ strata are in a relatively good condition to bear the cost of transportation. Regarding the treatment cost, Figure 3b indicates that inequality was more pronounced in 2010 compared to 2005. For instance, people at the 4 ^th^ & 5 ^th^ quintile of the economic strata are in relatively good position to afford the cost of treatment than in 2005 for all the instances of treatment. Figure 3c shows that for all instances of treatments, the total cost is most unequal in 2005 compared to 2010. For instance, those at the 4 ^th^ & 5 ^th^ quintiles of the economic strata are more able to bear total treatment costs relative to those at the lower strata.

## Discussion

This study provides the first detailed trend analysis of catastrophic OOP health expenditure and fairness of healthcare facilities utilization in Cambodia in 2005 and 2010. The findings show that the trend of catastrophic spending and fairness in utilization of health care facilities is not improving. The finding that more people from the poorer households are seeking treatment in all three instances compared to richer households over the two periods (2005 and 2010) is not new. The reason for this may be because individuals from lower strata of the economic index tend to patronize hawkers (here, a kind of quack without any medical degree selling medicine), as these providers are known to charge nominal fees ^24, 25^. Care seeking from hawkers and unqualified health care professionals has been shown to be a precursor for treatment failures and switching of care providers at private and public health facilities ^24, 26, 27^. The present finding supports a report from elsewhere in South East Asia, i.e. that poorer individuals have large healthcare burdens ^28^.

The present results displayed a huge increase in utilization of private facilities compared to public facilities from 2005 to 2010. Hence, one may argue that costs are an important determinant of choice of place of treatments. People would use more public facilities to avoid healthcare burden at private facilities. In this study, however, the trend in use of health care facilities has been shown to tilt consistently towards the use of private facilities for all instances of service use for the two time periods. This finding is not new, but is consistent with what has been reported before in other LMICs, and South East Asia in particular ^4, 28^. Countries in South East Asia have witnessed an increase in the proliferation of private health care facilities in the last decade. One probable reason for the present observed finding may be one of the unique features of poor health systems, where private and public care facilities always substitute each other, mostly in LMICs ^29^. In addition to this, inefficiency in service delivery in public health systems has been shown to be another contributory factor. For example, long waiting times, nonchalant attitudes of health care workers and perennial lack of essential medicines, coupled with informal fees charged by medical personnel to survive poor wages ^30– 32^.

Financing health care through formal and informal OOP payments have proven to affect people’s behavior in seeking care, especially those from poor households ^27, 33^. In this analysis, the pattern of OOP has been shown to be consistent and comprised of loans both with and without interests, wages/pocket money and savings in both 2005 and 2010. This finding supports what has been reported in several studies conducted across different countries ^34– 36^. The present analysis documents a wide variation in the mode of payment for health care between the two periods in relation to use of personal savings and wages/pocket money. Evidence has consistently shown that an individual from a poorer household faces a huge economic burden from health care costs than their richer counterpart ^36^. People saved more in 2010 and relied less on their wages and pocket money to offset payments for their treatment compared to 2005. Another explanation for this observation could be related to people’s awareness of the importance of setting aside a smaller portion of their income for health care emergencies to avoid selling and borrowing.

Health equity funds, exemption schemes and other subsidizations have been introduced to address inequity of health care utilization, which target the poor, in several LMICs. It can be difficult to identify and target the intended people for these exemption schemes and other subsidizations; however, studies in Cambodia found that the health equity fund to be rather successful in this country ^17^. In contrast, in the present study, the health equity fund only accounted for 2% of the OOP payments being made. Possession of health insurance has been shown to hold promise for financing individual health care costs in LMICs ^37^. The use of health insurance as a mode of payment for health service utilization was reported in 2010, but not in 2005. This finding is in line with the results from other studies ^38, 39^.

Economic hardship experienced by people from poorer households when accessing health care is immeasurable. As in other studies, the present analysis further documents the existence of inequity and unfairness in cost burden between the poor and the rich in Cambodia when accessing care. In summary, the distribution of cost burden and catastrophic spending among Cambodian adults was more inequitable over the two time periods. However, there is a growing understanding of the need to bridge poor-rich inequalities in access to health care through adoption of several coping mechanisms among the entire populace.

A health system should, according to the WHO, improve the health of the population they serve, respond to people’s expectations and provide financial protection against the costs of ill-health ^2^. With the present reported result, Cambodian health systems, which heavily rely on OOP payments, do not meet the last objective. Furthermore, according to the Strategic Plan 2008–2015 from the Ministry of Health, one of five working principles are “social health protection, especially for the poor and vulnerable groups” ^15^. The findings from this analysis echoes the need to ensure that those from poorer households are protected against hardship spending when seeking care. As such, more work is needed to guarantee access to sufficient health care for the poor and vulnerable in Cambodia. One of the ways to mitigate financial hardships constantly being faced by those from poorer households and reduce poverty drift through catastrophic health care spending is to adopt point-of-care health financing mechanisms targeted at those in need ^40^.

### Study limitations and strengths

This study had several important limitations that warrant mentioning. First, this analysis is based on existing survey data; hence it is difficult to ascertain the long-term effect of borrowing on individuals that may lead to economic hardship. Second, it is difficult to account for multiple modes of payments for health care use at individual levels. Third, this analysis used an asset-based index as a proxy measure of economic ability at household level and may be subject to criticism. However, an asset–based index as a surrogate measure for household wealth has been shown to be the most appropriate in LMICs ^20^.

### Policy implication

Findings from this analysis corresponds with the results of other studies and asserts the need for governments in LMICs to adopt a pro-poor funding mechanism. Although the introduction of an health equity fund is a welcome idea, Cambodian policy makers should take a cue from other LMICs and adopt multiple means tested health care financing option, such as community based health insurance and social insurance, to help mitigate financial hardship due to OOP payments ^41– 43^. In addition, the government should increase spending on services being provided at public health care facilities to reduce ever increasing reliance on private sector providers. These approaches would go a long way to reduce the economic burden of care utilization among the poorest.

## Data availability

The DHS Program owns data used in this study. The DHS for Cambodia 2005 and 2010 are available for researchers interested in further analyses. Researchers should contact the DHS Program and to get permission to use the required data ( https://dhsprogram.com/data/Access-Instructions.cfm).
