# Supplementary material for: Out-of-pocket health expenditure and fairness in utilization of health care facilities in Cambodia in 2005 and 2010
Source: F1000Res. 2017 Nov 29;6:2066. [Version 1] doi: 10.12688/f1000research.12801.1 (PMC5754743; doi:10.12688/f1000research.12801.1)
Supplement: Peer review history

# Peer review: Reviewer response for version 1

**DOI:** 10.5256/f1000research.13871.r28781

**Published:** 2018-01-04

**Authors:** Aziz Rahman

**Affiliations:** 1 Austin Health Clinical School of Nursing, La Trobe University, Melbourne, VIC, Australia; 2 Indigenous Health Equity Unit, The University of Melbourne, Parkville, VIC, Australia; 3 National Centre for Farmer Health, Deakin University, Geelong, VIC, Australia; 4 Institute for Breathing and Sleep (IBAS), Austin Health, Heidelberg, VIC, Australia

Findings of this study add further evidence regarding the burden of healthcare expenditure amongst the poor people in a developing country like Cambodia.

 The authors have explained the rationale for conducting this study well and utilized secondary database to answer the research question. Data comparison between 2010 and 2005 in the Figure and Table were informative. It was good to observe the changes regarding use of other facilities in the third instance in 2010 compared to the data from 2005. However, use of private facilities in the second instance did not change over years, which was not an unexpected scenario in developing countries although not acceptable. Hence policy makers should address the factors to increase utilization of public healthcare facilities. The major portion of the out of pocket expenditure was from either savings or wages/pocket money, which did not change over years as well.

 The authors have nicely indicated policy implications of the evidence generated from this study, which should be communicated to the policy makers in Cambodia to expect changes in future.

Is the work clearly and accurately presented and does it cite the current literature?

Yes

If applicable, is the statistical analysis and its interpretation appropriate?

I cannot comment. A qualified statistician is required.

Are all the source data underlying the results available to ensure full reproducibility?

Yes

Is the study design appropriate and is the work technically sound?

Yes

Are the conclusions drawn adequately supported by the results?

Yes

Are sufficient details of methods and analysis provided to allow replication by others?

Yes

Reviewer Expertise:

NA

I confirm that I have read this submission and believe that I have an appropriate level of expertise to confirm that it is of an acceptable scientific standard.

# Peer review: Reviewer response for version 1

**DOI:** 10.5256/f1000research.13871.r28958

**Published:** 2017-12-12

**Authors:** Kazi Selim Anwar

**Affiliations:** 1 Global Health Security Agenda (GHSA) Project, Institute of Epidemiology, Disease Control and Research (IEDCR), Dhaka, Bangladesh

Findings of this important study highlighted that the Combodian Govt. needs effective financial protection plan to promote spending on health-care-utilization. It would reduce economic burden of health-care utilization among the poorest communities. This research has high potential to allow a platform of replicating this study by others in LMICs.

Is the work clearly and accurately presented and does it cite the current literature?

Yes

If applicable, is the statistical analysis and its interpretation appropriate?

Yes

Are all the source data underlying the results available to ensure full reproducibility?

Yes

Is the study design appropriate and is the work technically sound?

Yes

Are the conclusions drawn adequately supported by the results?

Yes

Are sufficient details of methods and analysis provided to allow replication by others?

Yes

Reviewer Expertise:

NA

I confirm that I have read this submission and believe that I have an appropriate level of expertise to confirm that it is of an acceptable scientific standard.
